# P-1652. Geographical factors associated with antibiotic use for non-bacterial upper respiratory infections using nationwide Japanese data

**DOI:** 10.1093/ofid/ofae631.1818

**Published:** 2025-01-29

**Authors:** Taito Kitano, Shinya Tsuzuki, Ryuji Koizumi, Kensuke Aoyagi, Yusuke Asai, Yoshiki Kusama, Norio Ohmagari

**Affiliations:** AMR Clinical Reference Center, National Center for Global Health and Medicine, Shinjuku, Tokyo, Japan; National Center for Global Health and Medicine, Shinjuku-ku, Tokyo, Japan; National Center for Global health and Medicine, Shinjuku-ku, Tokyo, Japan; AMR Clinical Reference Center, National Center for Global Health and Medicine, Shinjuku, Tokyo, Japan; National Center for Global Health and Medicine, Shinjuku-ku, Tokyo, Japan; Tohoku University School of Medicine, Shinjuku-ku, Tokyo, Japan; National Centre for Global Health and Medicine, Shinjuku, Tokyo, Japan

## Abstract

**Background:**

The large number of antibiotic prescriptions for non-bacterial upper respiratory infections has been reported globally. The reduction of unnecessary antibiotic use for non-bacterial upper respiratory infections is critical. Elucidating geographical factors associated with antibiotic use for non-bacterial upper respiratory infections using nationwide data may guide national policy regarding antibiotic stewardship.

**Figure:**
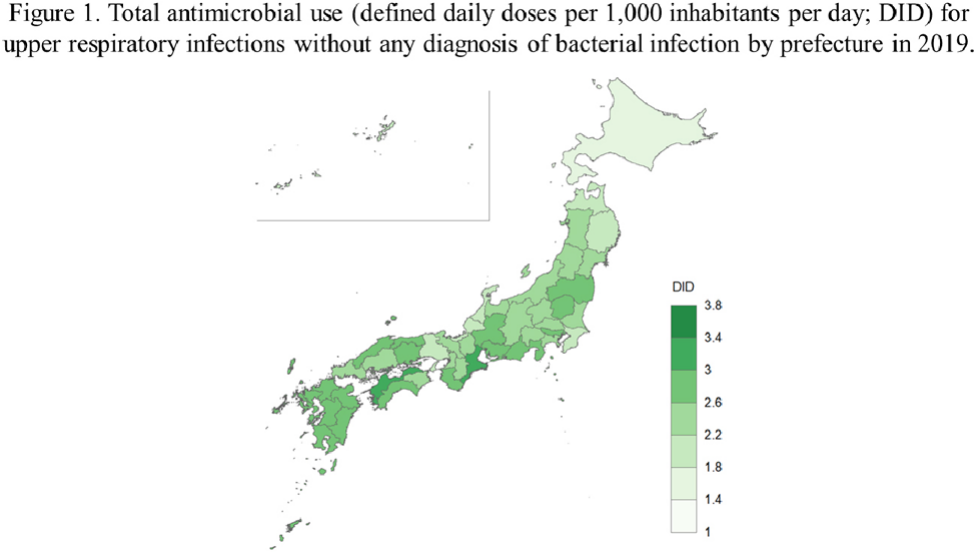

**Methods:**

This study evaluated geographical factors regarding antibiotic use for patients with sole diagnosis of upper respiratory infections using health insurance claims data across all Japanese prefectures. A multivariable negative binomial regression analysis was implemented for the outcome of the defined daily doses per 1,000 habitants per day by prefecture with patient- and physician-level variables. Total and sub-class antibiotic use were analyzed.

**Figure:**
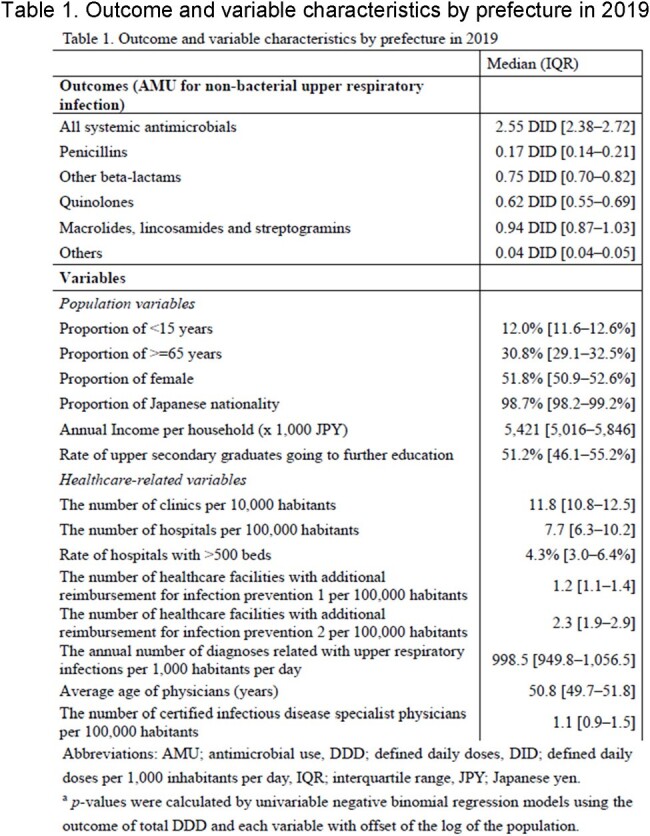

**Results:**

In 2019, 116,174,509 defined daily doses of antibiotic use for non-bacterial upper respiratory infections were included from all 47 prefectures. The annual number of diagnoses related with upper respiratory infections per 1,000 inhabitants per day (adjusted rate ratio [aRR] 1.001, [95% confidence interval 1.000-1.001], *p*=0.001) were significantly correlated with total antibiotic use. The mean age of physician (aRR 1.059 [1.009-1.113], *p*=0.019) for third generation cephalosporins and aRR 1.065 [1.011-1.122], *p*=0.016) for fluoroquinolones and the proportion of children age under 15 years (aRR 0.920 [0.876-0.966], *p*< 0.001) for third generation cephalosporins and aRR 0.926 [0.879-0.977], *p*=0.002 for fluoroquinolones) was also associated with the consumption of third generation cephalosporins and fluoroquinolones.

**Figure:**
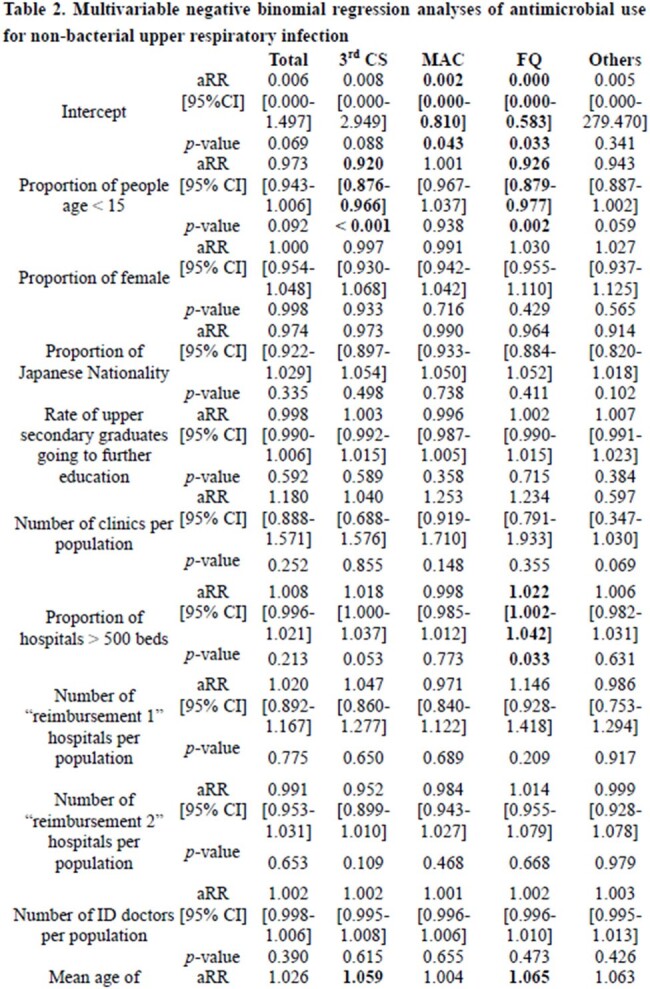

**Conclusion:**

This ecological study demonstrated that the proportion of children and the mean age of physicians were associated with the use of third generation cephalosporins and quinolones for non-bacterial upper respiratory infections. The results may contribute to the further development of the national stewardship strategies to reduce unnecessary antibiotic use for upper respiratory infections.

**Disclosures:**

**All Authors**: No reported disclosures

